# Systematic Review and Meta-Analysis of the Effect of Statins on Circulating E-Selectin, L-Selectin, and P-Selectin

**DOI:** 10.3390/biomedicines9111707

**Published:** 2021-11-17

**Authors:** Angelo Zinellu, Arduino A. Mangoni

**Affiliations:** 1 Department of Biomedical Sciences, University of Sassari, 07100 Sassari, Italy; azinellu@uniss.it; 2 Discipline of Clinical Pharmacology, College of Medicine and Public Health, Flinders University, Bedford Park, SA 5042, Australia; 3 Department of Clinical Pharmacology, Flinders Medical Centre, Southern Adelaide Local Health Network, Bedford Park, SA 5042, Australia

**Keywords:** statins, E-Selectin, L-Selectin, P-Selectin, atherosclerosis

## Abstract

The pleiotropic effects of statins might involve preventing inflammatory cell adhesion to the endothelium, which is a critical step in the pathogenesis of atherosclerosis. We conducted a systematic review and meta-analysis of the effects of statins on the circulating cell adhesion molecules E-Selectin, L-Selectin, and P-Selectin. A literature search was conducted in PubMed, Web of Science, and Scopus, from inception to July 2021. Risk of bias and certainty of evidence were assessed using the Joanna Briggs Institute Critical Appraisal Checklist and GRADE, respectively. In 61 studies, statins significantly reduced P-selectin (standard mean difference, SMD = −0.39, 95% CI −0.55 to −0.22, *p* < 0.001; moderate certainty of evidence), L-selectin (SMD = −0.49, 95% CI −0.89 to −0.10, *p* = 0.014; very low certainty of evidence), and E-Selectin (SMD = −0.73, 95% CI −1.02 to −0.43, *p* < 0.001; moderate certainty of evidence), independently of baseline lipid profile and other study and patient characteristics. The corresponding pooled SMD values in sensitivity analysis were not substantially altered when individual studies were sequentially removed. Simvastatin had a significant lowering effect on both P-selectin and E-selectin. Therefore, statins significantly reduce circulating selectins. Further studies are required to investigate whether selectin lowering mediates cardiovascular risk reduction with these agents. (PROSPERO registration number: CRD42021282778).

## 1. Introduction

A critical step in the pathophysiology of atherosclerosis involves the adhesion of inflammatory cell types to the endothelium. This process is facilitated by several cell adhesion molecules [1]. The selectins are a key family of cell adhesion molecules that includes the C-type lectins P-selectin, stored in platelets and endothelial cells [2]; L-selectin, expressed in leukocytes [3]; and E-selectin, expressed in the endothelium [2,4]. L-selectin mediates lymphocyte rolling, whereas P-selectin and E-selectin are primarily expressed in states of endothelial inflammation and facilitate monocyte, neutrophil, and lymphocyte rolling [4]. E-Selectin, L-Selectin, and P-Selectin also exist in soluble forms and can be measured in blood to characterize the state of endothelial and platelet activation in atherosclerosis [4,5,6].

The pathophysiological role of selectins, particularly P-Selectin and E-selectin, in atherosclerotic cardiovascular disease is supported both by experimental and human studies [7]. In particular, epidemiological studies have reported significant and positive associations between the concentration of soluble selectins and adverse cardiovascular outcomes. For example, higher soluble P-selectin concentrations have been shown to be significantly associated with incident cardiovascular events in women [8]. Similar associations between P-selectin and cardiovascular events have been reported in other studies [9,10]. Soluble E-selectin has also shown significant associations with incident cardiovascular disease in patients with renal failure [11] and atrial fibrillation [12].

Therefore, the available evidence suggests that soluble selectins play a critical role in the pathophysiology of atherosclerosis and endothelial dysfunction and as markers of cardiovascular risk [7], and that interventions targeting this family of cell adhesion molecules might exert atheroprotective effects [13].

Treatment with statins, the leading class of lipid-lowering agents for cardiovascular prevention [14,15], has been shown to exert beneficial effects on endothelial and vascular homeostasis independently of their primary target, inhibition of 3-hydroxy-3-methylglutaryl-CoA (HMG-CoA) reductase [16]. These so-called pleiotropic effects of statins include the suppression of specific pro-inflammatory cytokines with consequent reduced activation of selectins [17,18,19]. As human studies have investigated the effects of treatment with statins on soluble concentrations of selectins, we sought to critically appraise this evidence by conducting a systematic review and meta-analysis of the effects of statins on circulating E-Selectin, L-Selectin, and P-Selectin. Meta-regression and subgroup analyses were also conducted to investigate associations between effect size and specific study and patient characteristics.

## 2. Materials and Methods

A systematic literature search was conducted in the electronic databases PubMed, Web of Science, and Scopus, from inception to July 2021, using the following terms and their combination: “Selectin” or “P-Selectin” or “L-Selectin” or “E-Selectin” and “Statin”. Abstracts were independently screened by two investigators. If relevant, the full-text articles were reviewed according to the following eligibility criteria: (1) assessment of soluble P-Selectin and/or L-Selectin and/or E-Selectin in plasma or serum at baseline and after statin treatment; (2) ≥10 adult participants; (3) English language; and (4) full-text available. The references of the retrieved articles were also searched to identify additional studies. Any disagreement between reviewers was resolved by a third investigator. Data extracted from each study included the year of publication; the continent where the study was conducted; age; the proportion of males; the concentrations of P-Selectin, L-Selectin, and E-Selectin before and after treatment; the primary condition studied; baseline lipid profile; statin and daily dose used; and treatment duration.

The risk of bias was assessed using the Joanna Briggs Institute (JBI) Critical Appraisal Checklist for analytical studies. A score of ≥5, 4, and <4 indicated low, moderate, and high risk, respectively [20]. The certainty of evidence was assessed using the Grades of Recommendation, Assessment, Development and Evaluation (GRADE) Working Group system. GRADE considers the study design (randomized vs. observational), the risk of bias (JBI checklist), the presence of unexplained heterogeneity, the indirectness of evidence, the imprecision of results (sample size, 95% confidence interval width and threshold crossing), the effect size (small, SMD < 0.5, moderate, SMD 0.5–0.8, and large, SMD >0.8) [21], and the probability of publication bias [22,23,24]. The study complied with the Preferred Reporting Items for Systematic reviews and Meta-Analyses (PRISMA) 2020 statement (Supplementary Materials, Tables S1 and S2) [25]. The protocol was registered in the International Prospective Register of Systematic Reviews (PROSPERO registration number: CRD42021282778).

### Statistical Analysis

Standardized mean differences (SMDs) were calculated to generate forest plots of continuous data and to evaluate differences in selectin serum concentrations before and after statin treatment. If necessary, means and standard deviations were extrapolated from medians and interquartile ranges [26], or from graphs using the Graph Data Extractor software. Heterogeneity of SMD across studies was assessed using the Q-statistic (significance level set at *p* < 0.10) and I^2^-statistic (<25%, no heterogeneity; between 25–50%, moderate heterogeneity; between 50–75%, large heterogeneity; and >75%, extreme heterogeneity) [27,28]. Random-effects models were used in the presence of significant heterogeneity (I^2^ ≥ 50%). Sensitivity analysis was conducted to investigate the influence of each study on the overall risk estimate [29]. The presence of publication bias was assessed using the Begg’s and Egger’s tests (significance level set at *p* < 0.05) [30,31], and the Duval and Tweedie “trim-and-fill” method [32].

Univariate meta-regression analyses were conducted to investigate associations between effect size and the following study and patient characteristics: age; proportion of males; body mass index; baseline total cholesterol, low-density lipoproteins (LDL)-cholesterol, high-density lipoproteins (HDL)-cholesterol, and triglycerides; year of publication; sample size; continent where the study was conducted; specific statin and class used (lipophilic: atorvastatin, simvastatin, lovastatin, fluvastatin, cerivastatin, and pitavastatin; hydrophilic: rosuvastatin, pravastatin); and treatment duration. Pre-planned subgroup analyses investigated the effects of specific statins, statin classes, and continent where the study was conducted. Statistical analyses were performed using Stata 14 (STATA Corp., College Station, TX, USA).

## 3. Results

### 3.1. Systematic Research

**Figure 1:**
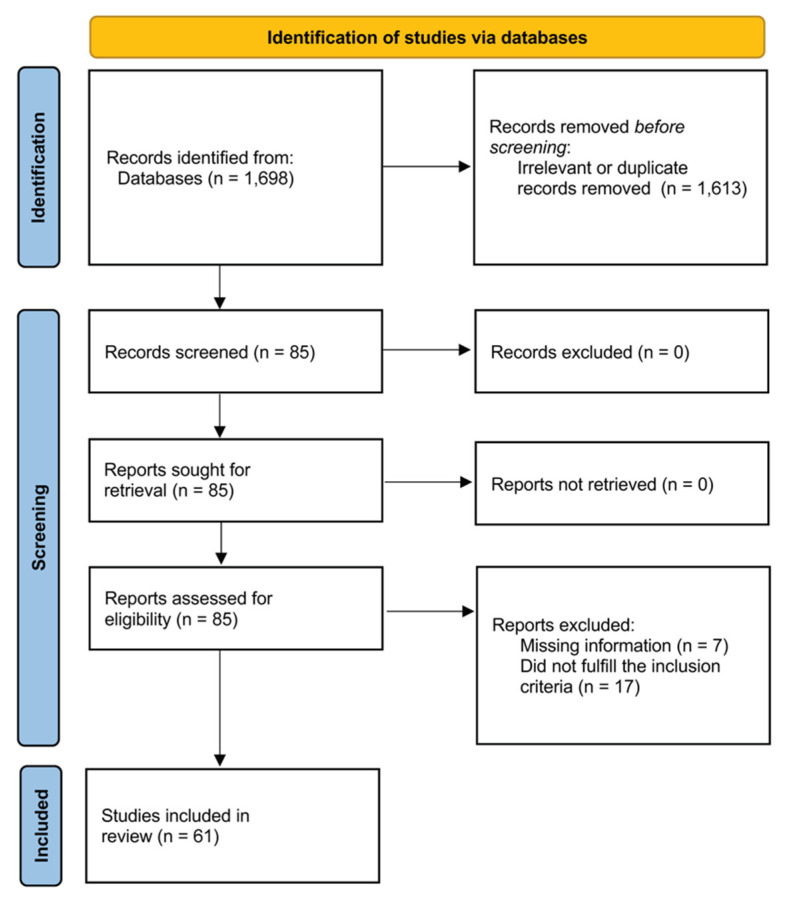
PRISMA 2020 flow chart.

A flow chart describing the screening process is presented in Figure 1. We initially identified 1698 articles. A total of 1613 were excluded after the first screening because they were either duplicates or irrelevant. After a full-text review of the remaining 85 articles, 24 were further excluded due to missing data (n = 7) or because they did not fulfil the inclusion criteria (n = 1, age < 18 years; n = 4, participants already on lipid-lowering treatment; n = 3, sample size < 10; n = 9 measurement of cell surface selectin). Thus, 61 studies published between 1999 and 2018 were included in the final analysis (Table 1) [33,34,35,36,37,38,39,40,41,42,43,44,45,46,47,48,49,50,51,52,53,54,55,56,57,58,59,60,61,62,63,64,65,66,67,68,69,70,71,72,73,74,75,76,77,78,79,80,81,82,83,84,85,86,87,88,89,90,91,92,93].

### 3.2. Meta-Analysis of Soluble P-Selectin

#### 3.2.1. Study Characteristics

Thirty-three studies reported 41 treatment arms in 1238 participants (mean age 59 years, 55% males) [34,35,38,40,43,44,47,49,50,52,56,58,59,60,62,63,64,65,66,68,70,71,72,74,76,78,80,81,82,84,87,90,93]. Simvastatin was used in 17 arms [47,52,56,58,59,60,62,66,78,81,82,90,93], atorvastatin in 9 [43,44,63,70,72,74,80,87], pravastatin in 7 [34,38,40,65,84], fluvastatin in 3 [35,49,50], pitavastatin in 3 [68,76], and rosuvastatin [71] and combination of various statins [64] in 1, respectively. Treatment duration ranged between 2 and 24 weeks (Table 1).

#### 3.2.2. Risk of Bias

The risk of bias was low in all studies [34,35,38,40,43,44,47,49,50,52,56,58,59,60,62,63,64,65,66,68,70,71,72,74,76,78,80,81,82,84,87,90,93] (Table 2).

#### 3.2.3. Results of Individual Studies and Syntheses

**Figure 2:**
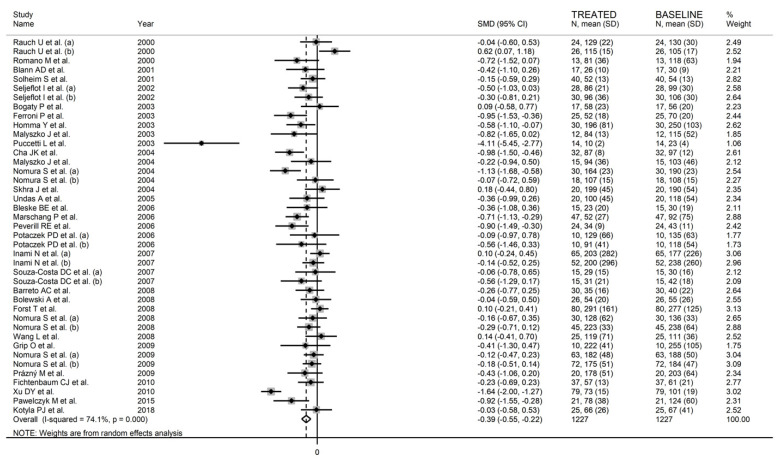
Forest plot of circulating P-Selectin concentrations before and after statin treatment.

The forest plot for circulating P-Selectin concentrations before and after statin treatment is shown in Figure 2. In six arms [34,44,60,68,74,78], concentrations were higher after treatment (mean difference range, 0.10 to 0.62); however, the difference was statistically significant only in one [34]. In the remaining arms [35,38,40,43,47,49,50,52,56,58,59,62,63,64,65,66,70,71,72,76,80,81,82,84,87,90,93], P-Selectin concentrations were lower after treatment (mean difference range, −0.03 to −4.11) with a significant difference reported in nine [47,49,52,56,59,64,65,87,90]. The large between-study heterogeneity observed (I^2^ = 74.1%, *p* < 0.001) prompted the use of random-effects models. Pooled results showed that P-Selectin concentrations were significantly lower after statin treatment (SMD = −0.39, 95% CI −0.55 to −0.22, *p* < 0.001).

**Figure 3:**
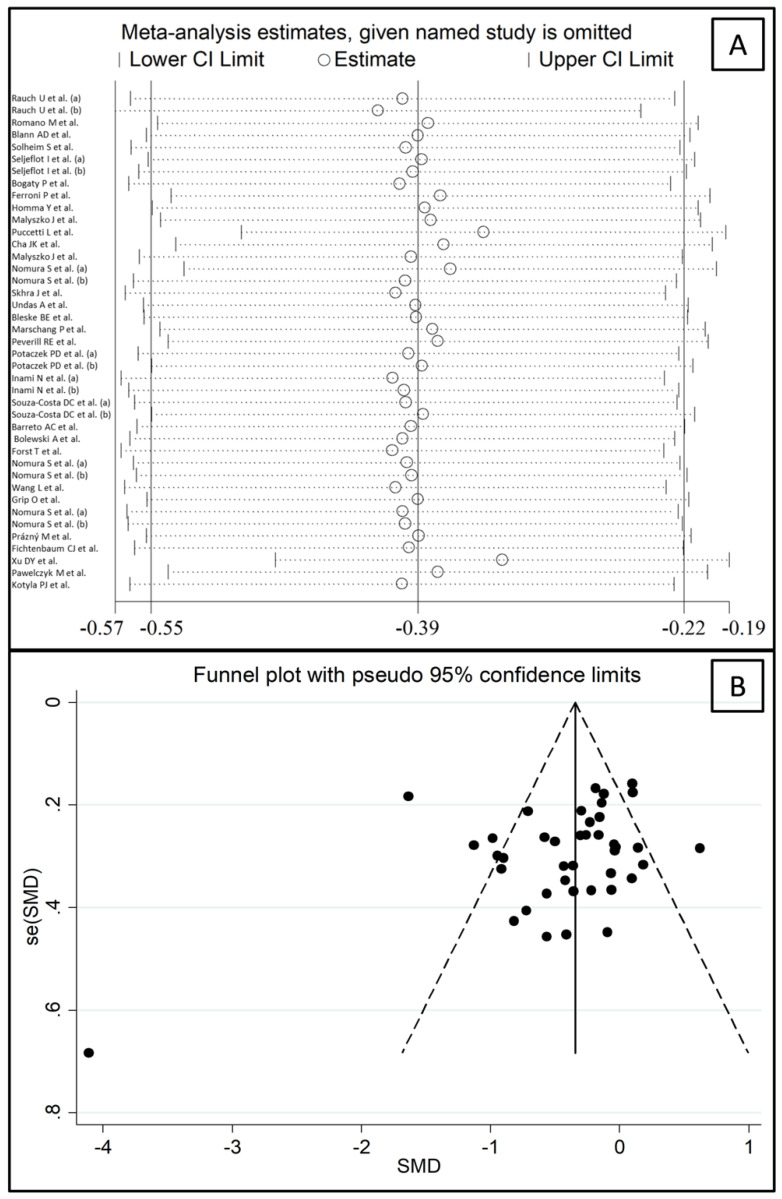
(**A**) Sensitivity analysis of the association between P-Selectin and statin treatment. The influence of individual studies on the overall standardized mean difference (SMD) is shown. The middle vertical axis indicates the overall SMD, and the two vertical axes indicate the 95% confidence intervals (CIs). The hollow circles represent the pooled SMD when the remaining study is omitted from the meta-analysis. The two ends of each broken line represent the 95% CIs. (**B**) Funnel plot of studies investigating P-Selectin concentrations before and after statin treatment.

In sensitivity analysis, the corresponding pooled SMD values were not substantially altered when individual studies were sequentially removed (effect size range, between −0.41 and −0.33, Figure 3A). However, the funnel plot analysis, reported in Figure 3B, detected a distortive effect of one study [52]. Removing this study mildly attenuated both the effect size (SMD = −0.35, 95% CI −0.50 to −0.20, *p* < 0.001) and the heterogeneity (I^2^ = 68.5%, *p* < 0.001).

#### 3.2.4. Publication Bias

**Figure 4:**
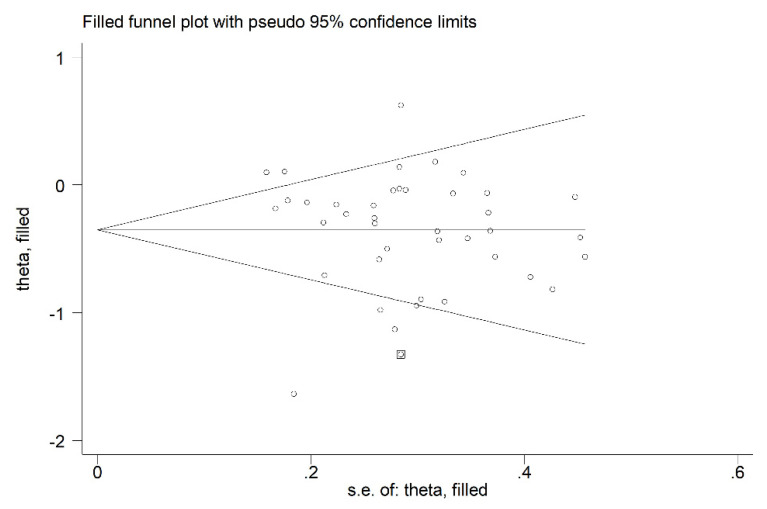
Funnel plot of P-Selectin concentrations before and after statin treatment after “trimming-and-filling”. Dummy studies and genuine studies are represented by enclosed circles and free circles, respectively.

The analysis of the remaining 40 arms, after removing the previously described study [52], did not show significant publication bias (Begg’s test, *p* = 0.12; Egger’s test, *p* = 0.57). The “trim-and-fill” method identified one potential missing study to be added to the left side of the funnel plot to ensure symmetry (adjusted SMD = −0.37, 95% CI −0.52 to −0.22, *p* < 0.001; Figure 4).

#### 3.2.5. Meta-Regression and Subgroup Analysis

**Figure 5:**
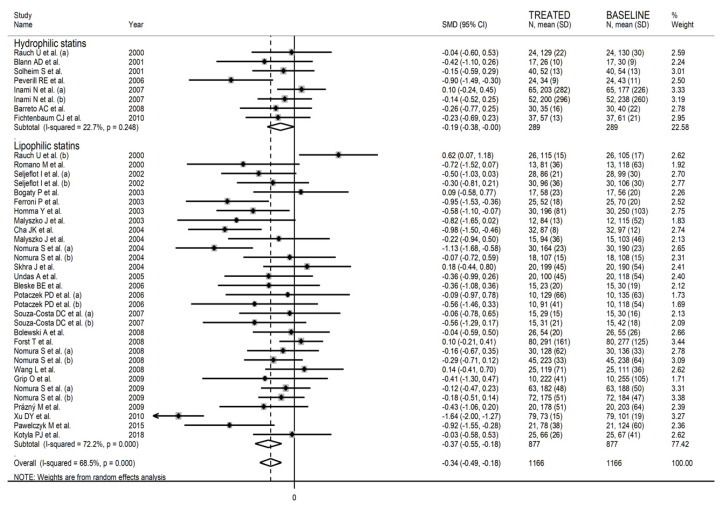
Forest plot of P-Selectin concentrations before and after statin treatment according to statin class (hydrophilic or lipophilic).

In univariate meta-regression, there were no significant associations between effect size and age (t = −0.61, *p* = 0.55); proportion of males (t = 0.03, *p* = 0.98); body mass index (t = 0.43, *p* = 0.67); publication year (t = −0.37, *p* = 0.71); sample size (t = −0.23, *p* = 0.81); baseline total cholesterol (t = 0.80, *p* = 0.43), LDL-cholesterol (t = 0.95, *p* = 0.35), HDL-cholesterol (t = 0.36, *p* = 0.72), and triglycerides (t = 0.79, *p* = 0.44); and treatment duration (t = 0.19, *p* = 0.85). In sub-group analysis, the P-Selectin-lowering effect with lipophilic statins (SMD = −0.37, 95% CI −0.55 to −0.18, *p* < 0.001; I^2^ = 72.2%, *p* < 0.001) was relatively larger than that with hydrophilic statins (SMD = −0.19, 95% CI −0.38 to −0.004, *p* = 0.046; I^2^ = 22.7%, *p* = 0.25, Figure 5); however, this difference was not statistically significant (*p* = 0.45).

**Figure 6:**
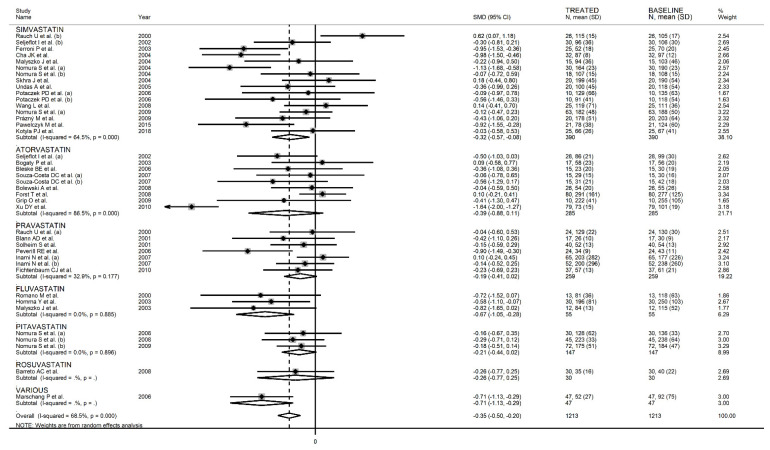
Forest plot of P-Selectin concentrations before and after statin treatment according to individual agents.

**Figure 7:**
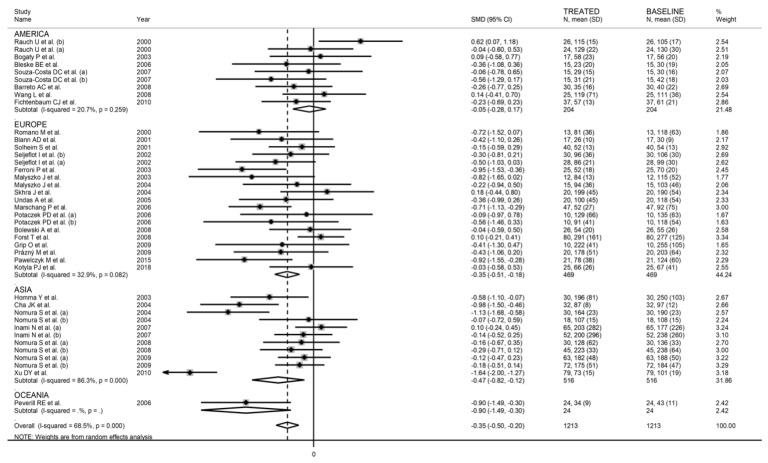
Forest plot of P-Selectin concentrations before and after statin treatment according to continent where the study was conducted.

When considering individual agents, a significant lowering effect was observed with simvastatin (SMD = −0.32, 95% CI −0.57 to −0.08, *p* = 0.005; I^2^ = 64.5%, *p* < 0.001) and fluvastatin (SMD = −0.67, 95% CI −1.05 to −0.28, *p* = 0.001; I^2^ = 0.0%, *p* = 0.89), but not with atorvastatin (SMD = −0.39, 95% CI −0.88 to 0.11, *p* = 0.12; I^2^ = 86.5%, *p* < 0.001), pravastatin (SMD = −0.19, 95% CI −0.41 to 0.02, *p* = 0.06; I^2^ = 32.9%, *p* = 0.18), or pitavastatin (SMD = −0.21, 95% CI −0.44 to 0.02, *p* = 0.07; I^2^ = 0.0%, *p* = 0.98) (Figure 6). As reported in Figure 7, a significant P-Selectin-lowering effect was reported in studies conducted in Asia (SMD = −0.47, 95% CI −0.82 to −0.12, *p* = 0.008; I^2^ = 86.3%, *p* < 0.001) and Europe (SMD = −0.35, 95% CI −0.51 to −0.18, *p* < 0.001; I^2^ = 32.9%, *p* = 0.08) but not America (SMD = −0.05, 95% CI −0.28 to 0.17, *p* = 0.63; I^2^ = 20.7%, *p* = 0.26).

**Figure 8:**
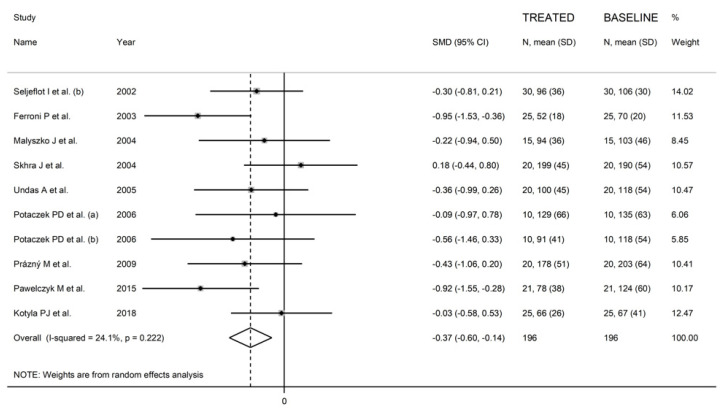
Forest plot of a sub-group of 10 studies examining P-Selectin concentrations, homogeneous for statin used and continent.

We further sought to identify more homogeneous study sub-groups according to statin used and continent. In a sub-group of nine studies (10 treatment arms) conducted in Europe using atorvastatin [43,47,58,60,62,66,82,90,93], the significant reduction in P-Selectin concentrations (SMD = −0.37, 95% CI −0.60 to −0.14, *p* = 0.002) was associated with a markedly lower heterogeneity (I^2^ = 24.1%, *p* = 0.22) (Figure 8).

#### 3.2.6. Certainty of Evidence

The initial level of certainty for P-Selectin SMD values was considered moderate because of the interventional nature of the studies (rating 3, ⊕⊕⊕⊝). After considering the low risk of bias in all studies (upgrade one level), a generally large heterogeneity was partially explained by the specific statin used and the continent where the study was conducted (no rating change required), the lack of indirectness (no rating change required), the relatively low imprecision (relatively narrow confidence intervals without threshold crossing, no rating change required), the relatively small effect size (SMD = −0.39, downgrade one level), and the absence of publication bias (no rating change required); the overall level of certainty remained moderate (rating 3, ⊕⊕⊕⊝).

### 3.3. Meta-Analysis of Soluble L-Selectin

#### 3.3.1. Study Characteristics

Four studies reported six treatment arms in 186 participants (mean age 55 years, 59% males) [34,42,76,93]. The statin used was simvastatin in two arms [34,93], pitavastatin in two [76], and atorvastatin [42] and pravastatin [34] in one, respectively. Duration of therapy ranged between 4 and 24 weeks (Table 1).

#### 3.3.2. Risk of Bias

The risk of bias was low in all studies [34,42,76,93] (Table 2).

#### 3.3.3. Results of Individual Studies and Syntheses

**Figure 9:**
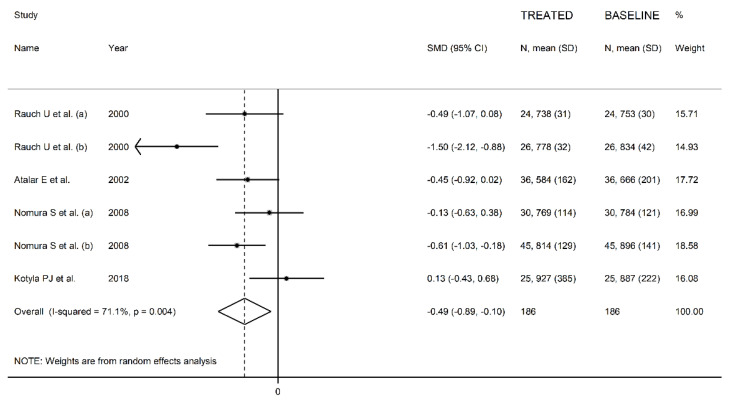
Forest plot of L-Selectin concentrations before and after statin treatment.

**Figure 10:**
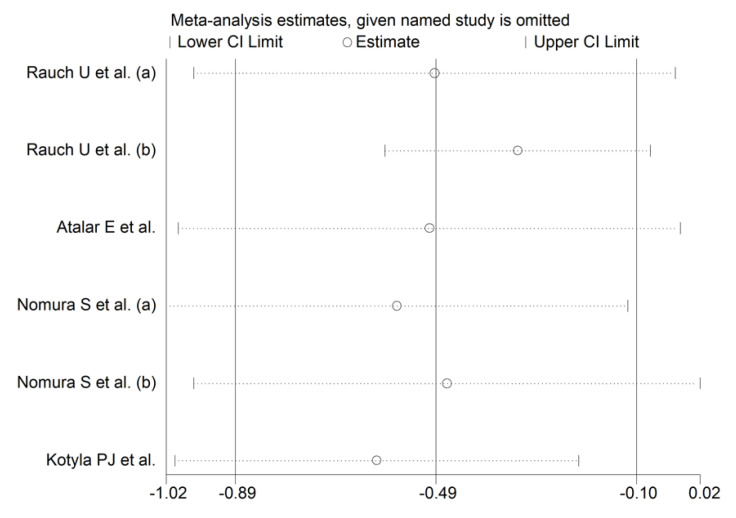
Sensitivity analysis of the association between L-Selectin and statin treatment. The influence of individual studies on the overall standardized mean difference (SMD) is shown. The middle vertical axis indicates the overall SMD, and the two vertical axes indicate the 95% confidence intervals (CIs). The hollow circles represent the pooled SMD when the remaining study is omitted from the meta-analysis. The two ends of each broken line represent the 95% CIs.

The forest plot for circulating L-Selectin concentrations before and after statin treatment is shown in Figure 9. In five arms [34,42,76], concentrations were lower after treatment (mean difference range, −1.50 to −0.13), and the difference was statistically significant in two [34,76]. In the remaining arm [93], L-Selectin concentrations were non-significantly higher after treatment. Random-effects models were used in view of the large heterogeneity observed (I^2^ = 71.1%, *p* = 0.004). Pooled results showed that L-Selectin concentrations were significantly lower after treatment (SMD = −0.49, 95% CI −0.89 to −0.10, *p* = 0.014). In sensitivity analysis, the corresponding pooled SMD values were not substantially altered when individual studies were sequentially removed (effect size range, between −0.61 and −0.33, Figure 10).

#### 3.3.4. Publication Bias

Assessment of publication bias was not possible because of the small number of studies.

#### 3.3.5. Meta-Regression and Subgroup Analysis

Meta-regression and sub-group analysis were not possible because of the small number of studies.

#### 3.3.6. Certainty of Evidence

The initial level of certainty for L-Selectin SMD values was considered moderate because of the interventional nature of the studies (rating 3, ⊕⊕⊕⊝). After considering the low risk of bias in all studies (upgrade one level), a large unexplained heterogeneity (downgrade one level), the lack of indirectness (no rating change required), the relatively low imprecision (relatively narrow confidence intervals without threshold crossing, no rating change required), the relatively small effect size (SMD = −0.49, downgrade one level), and the lack of assessment of publication bias (downgrade one level), the overall level of certainty was considered very low (rating 1, ⊕⊝⊝⊝).

### 3.4. Meta-Analysis of Soluble E-Selectin

#### 3.4.1. Study Characteristics

Thirty-eight studies reported 41 treatment arms in 1097 patients (mean age 55 years, 60% males) [33,36,37,39,40,41,43,44,45,46,48,51,53,54,55,57,58,60,61,64,67,69,72,73,75,76,77,79,80,82,83,85,86,88,89,91,92,93]. Simvastatin was used in 19 arms [33,36,37,39,43,53,54,55,57,58,60,61,73,82,83,85,86,91,92,93], atorvastatin in 15 [43,44,45,46,51,67,72,75,77,79,80,88,89], pravastatin in 2 [40,69], pitavastatin in 2 [76], various combination of statins in 2 [48,64], and fluvastatin in 1 [41]. Duration of therapy ranged between 4 and 52 weeks (Table 1).

#### 3.4.2. Risk of Bias

The risk of bias was low in all studies [33,36,37,39,40,41,43,44,45,46,48,51,53,54,55,57,58,60,61,64,67,69,72,73,75,76,77,79,80,82,83,85,86,88,89,91,92,93] (Table 2).

#### 3.4.3. Results of Individual Studies and Syntheses

**Figure 11:**
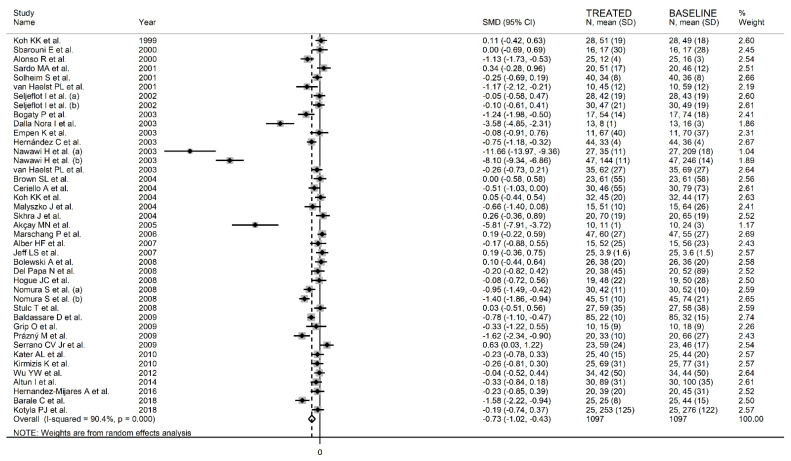
Forest plot of E-Selectin concentrations before and after statin treatment.

**Figure 12:**
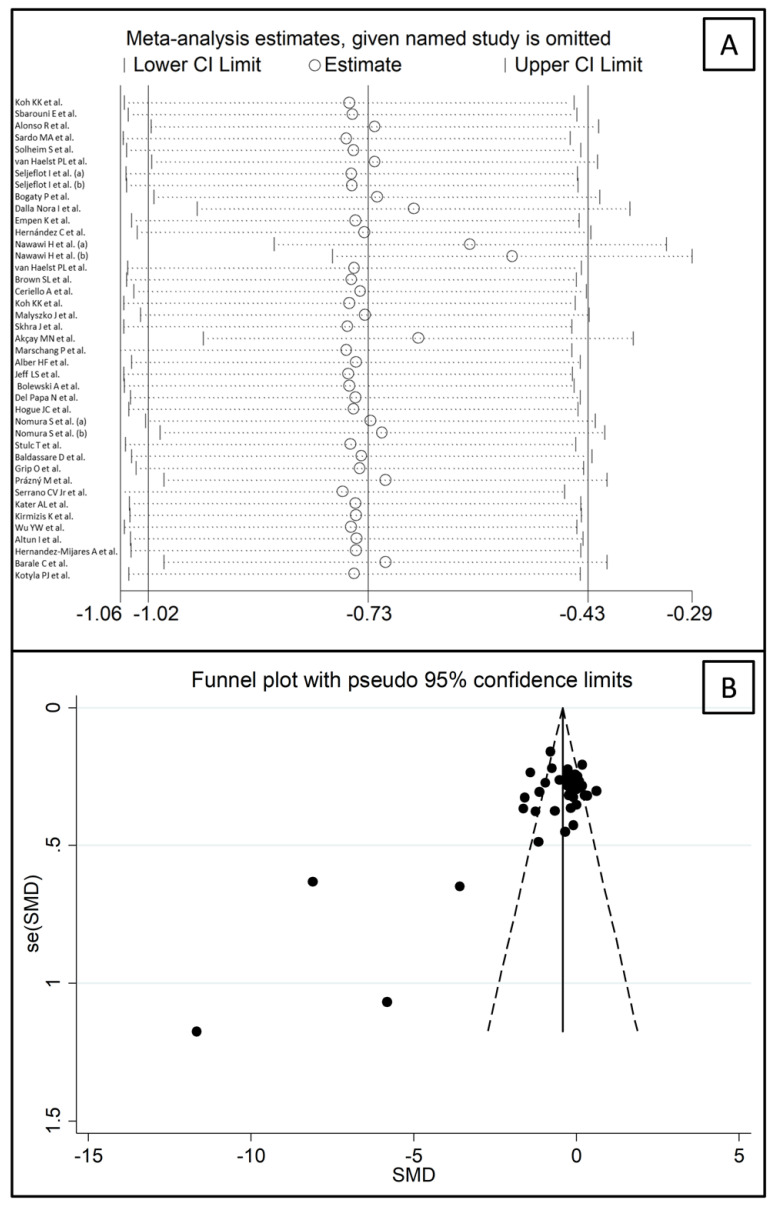
(**A**) Sensitivity analysis of the association between E-Selectin and statin treatment. The influence of individual studies on the overall standardized mean difference (SMD) is shown. The middle vertical axis indicates the overall SMD, and the two vertical axes indicate the 95% confidence intervals (CIs). The hollow circles represent the pooled SMD when the remaining study is omitted from the meta-analysis. The two ends of each broken line represent the 95% CIs. (**B**) Funnel plot of studies investigating E-Selectin concentrations before and after statin treatment.

The forest plot for circulating E-Selectin concentrations before and after statin treatment is shown in Figure 11. In nine arms [43,49,67,70,74,79,82,87,93], concentrations were higher after treatment (mean difference range, 0.03 to 0.63); however, a significant difference was reported only in one [83]. Virtually identical pre- and post-treatment concentrations were reported in two arms [36,54]. In the remaining arms [37,40,41,43,44,45,46,48,51,53,55,58,61,67,73,75,76,79,80,82,85,86,88,89,91,92,93], E-Selectin concentrations were lower after treatment (mean difference range, −0.04 to −11.66), with a significant difference reported in 13 [37,41,44,45,48,51,61,76,79,82,92]. Extreme heterogeneity between studies was observed (I^2^ = 90.4%, *p* < 0.001), requiring the use of random-effects models. Pooled results showed that circulating E-Selectin concentrations were significantly lower after treatment (SMD = −0.73, 95% CI −1.02 to −0.43, *p* < 0.001). In sensitivity analysis, the corresponding pooled SMD values were not substantially altered when individual studies were sequentially removed (effect size range, between −0.76 and −0.53, Figure 12A). However, the funnel plot analysis, reported in Figure 12B, detected a distortive effect of three studies (four treatment arms) [45,51,61]. Their removal attenuated both the effect size (SMD = −0.33, 95% CI −0.50 to −0.16, *p* < 0.001) and the magnitude of the heterogeneity (I^2^ = 71.4%, *p* < 0.001).

#### 3.4.4. Publication Bias

**Figure 13:**
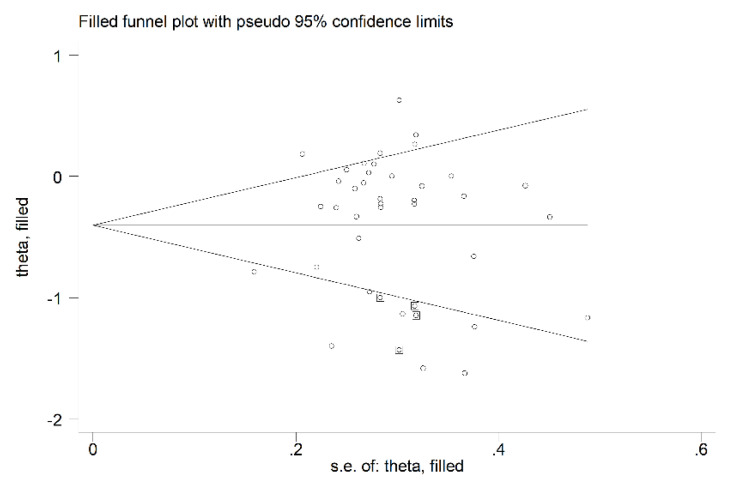
Funnel plot of studies investigating E-Selectin concentrations before and after statin treatment after “trimming-and-filling”. Dummy studies and genuine studies are represented by enclosed circles and free circles, respectively.

The analysis of the remaining 37 treatment arms did not show publication bias (Begg’s test, *p* = 0.57; Egger’s test, *p* = 0.90). However, the “trim-and-fill” method identified four potential missing studies to be added to the left side of the funnel plot to ensure symmetry (adjusted SMD = −0.41, 95% CI −0.58 to −0.24, *p* < 0.001; Figure 13).

#### 3.4.5. Meta-Regression and Subgroup Analysis

**Figure 14:**
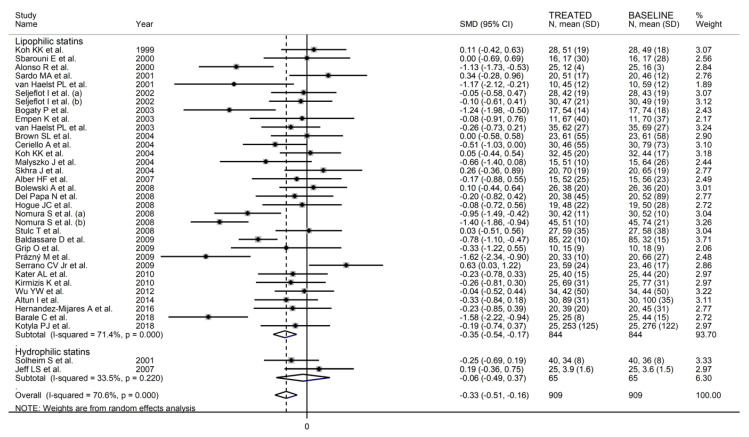
Forest plot of E-Selectin concentrations before and after statin treatment according to statin class (hydrophilic or lipophilic).

**Figure 15:**
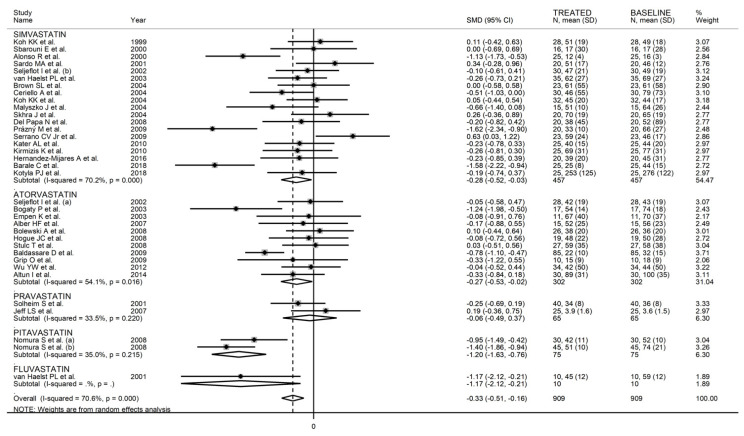
Forest plot of E-Selectin concentrations before and after statin treatment according to individual agents.

**Figure 16:**
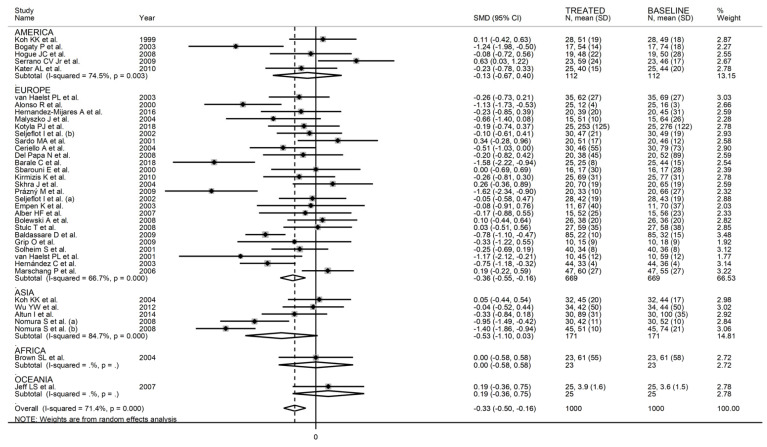
Forest plot of E-Selectin concentrations before and after statin treatment according to the continent where the study was conducted.

In univariate meta-regression, there were no significant associations between effect size and age (t = −0.23, *p* = 0.82), proportion of males (t = −0.58, *p* = 0.57), body mass index (t = 0.43, *p* = 0.67), publication year (t = −0.87, *p* = 0.31), sample size (t = −0.90, *p* = 0.38), baseline total cholesterol (t = −0.37, *p* = 0.71), LDL-cholesterol (t = −0.30, *p* = 0.77), HDL-cholesterol (t = 0.91, *p* = 0.37), and triglycerides (t = 0.94, *p* = 0.36), and treatment duration (t = −1.44, *p* = 0.16). In sub-group analysis, a significant lowering effect was observed with lipophilic (SMD = −0.35, 95% CI −0.54 to −0.17, *p* < 0.001; I^2^ = 71.4%, *p* < 0.001) but not hydrophilic statins (SMD = −0.06, 95% CI −0.49 to 0.37, *p* = 0.65; I^2^ = 33.5%, *p* = 0.22) (Figure 14). When assessing individual agents, a significant lowering effect was observed with simvastatin (SMD = −0.28, 95% CI −0.52 to −0.03, *p* = 0.03; I^2^ = 70.2%, *p* < 0.001), atorvastatin (SMD = −0.27, 95% CI −0.53 to −0.02, p = 0.035; I^2^ = 54.1%, *p* = 0.016), and pitavastatin (SMD = −1.20, 95% CI −1.63 to −0.76, *p* < 0.001; I^2^ = 35.0%, *p* = 0.22), but not pravastatin (SMD = −0.06, 95% CI −0.49 to 0.37, *p* = 0.65; I^2^ = 33.5%, *p* = 0.22) (Figure 15). Moreover, as reported in Figure 16, a significant decrease in E-Selectin concentrations was reported in studies conducted in Europe (SMD = −0.36, 95% CI −0.55 to −0.16, *p* < 0.001; I^2^ = 66.7%, *p* < 0.001) but not America (SMD = −0.13, 95% CI −0.67 to 0.40 *p* = 0.62; I^2^ = 20.7%, *p* = 0.26) or Asia (SMD = −0.53, 95% CI −1.10 to 0.03, *p* = 0.06; I^2^ = 84.7%, *p* < 0.001).

#### 3.4.6. Certainty of Evidence

The initial level of certainty for E-Selectin SMD values was considered moderate because of the interventional nature of the studies (rating 3, ⊕⊕⊕⊝). After considering the low risk of bias in all studies (upgrade one level), a large heterogeneity that was only partially attenuated after removing three studies (downgrade one level); the lack of indirectness (no rating change required); the relatively low imprecision (relatively narrow confidence intervals without threshold crossing, no rating change required); the relatively moderate effect size (SMD = −0.73, no rating change required); and the lack of publication bias (no rating change required), the overall level of certainty remained moderate (rating 3, ⊕⊕⊕⊝).

## 4. Discussion

In this systematic review and meta-analysis, statin treatment significantly reduced the concentrations of soluble E-Selectin, L-Selectin, and P-Selectin in participants with a range of cardiovascular risk profiles. In sensitivity analysis, the pooled SMD values were not substantially altered when individual studies were sequentially removed. In meta-regression, no significant associations were observed between effect size and various patient and study characteristics, including baseline lipids. The absence of significant associations with treatment duration, ranging between 2 and 52 weeks, suggests that the selectin-lowering effects of statins are evident relatively early during treatment and are maintained for up to 1 year.

The activation of selectins, particularly P-Selectin and E-Selectin, has an established role in the pathogenesis of atherosclerosis and its clinical manifestations [94]. The results of several observational studies further support this proposition. In the Women’s Health Study, participants with baseline soluble P-selectin concentrations in the highest quartile had a relative risk of suffering a cardiovascular event during a 3.5-year follow up period 2.2 times higher than those in the lowest quartile (95% CI 1.2 to 4.2). Notably, this association was independent of obesity, hypertension, hypercholesterolaemia, diabetes, and physical activity [8]. In another study in 733 patients undergoing coronary revascularization, those with baseline P-selectin concentrations in the second, third, and fourth quartile were at higher risk of experiencing a major cardiovascular event during a 9.7-year follow up period compared to the first quartile (hazard ratio, HR, 1.23, 95% CI 0.90 to 1.69; HR 1.48, 95% CI 1.08 to 2.02; and HR 1.57, 95% CI 1.11 to 2.15, respectively), after adjusting for confounders [9]. In a prospective study of 1041 adult patients with end-stage chronic kidney disease, each 0.1-log unit increase in P-selectin concentrations was significantly associated, after adjusting for confounders, with cardiovascular mortality (HR 1.10, 95% CI 1.02 to 1.27) and sudden cardiac death (HR 1.12, 95% CI 1.01 to 1.25) in males, but not in females, during a median follow-up of 38.2 months [10]. In a similar group of patients with end-stage renal disease, the risk of fatal and non-fatal cardiovascular events was significantly higher, after adjusting for confounders, in the highest vs. lowest tertile of soluble E-Selectin concentrations (HR 1.93, 95% CI 1.03 to 3.56) during a 21-month follow-up period [11]. Finally, in 423 patients with non-valvular atrial fibrillation and other cardiovascular risk factors followed for 19 months, those with soluble E-Selectin concentrations in the upper tertile had a significantly higher risk of adverse clinical events when compared to the bottom tertile (relative risk, RR, 3.70, 95% CI 2.51 to 5.31) [12].

The observed associations between soluble selectins and cardiovascular risk have stimulated the search for novel therapies that target these cell adhesion molecules. One of these agents, the monoclonal antibody against P-selectin inclacumab, has shown some promise in minimizing cardiac damage in patients with acute coronary syndrome undergoing percutaneous coronary intervention [95,96]. The results of our systematic review and meta-analysis suggest that soluble selectin-lowering might also be important in the context of statin therapy, an established treatment option in primary and secondary cardiovascular prevention [97,98,99]. Whist the exact mechanisms of action involved in the statin-mediated reduction in soluble selectin concentrations remain elusive, in vitro studies have shown that atorvastatin significantly prevents the overexpression of E-Selectin induced by cigarette smoking extract in human umbilical vein endothelial cells through inhibiting the NF-_K_B signal pathway, a critical pathway involved in inflammatory processes [100,101]. Treatment with simvastatin has been shown to prevent the release of the enzyme semicarbazide-sensitive amine oxidase/vascular adhesion protein 1, with consequent reduction of soluble E-Selectin [102]. Similar effects of simvastatin on the expression of P-Selectin and E-selectin have been reported in other studies [103]. Furthermore, treatment with atorvastatin significantly reduced the expression of P-Selectin in platelet-derived microparticles in patients with peripheral vascular disease [104].

In subgroup analysis, lipophilic, but not hydrophilic, statins significantly reduced soluble E-Selectin concentrations. However, these results need to be interpreted with caution because of the extremely low number of treatment arms, two, involving hydrophilic statins. Significant differences were also observed with individual statins, with simvastatin and fluvastatin being particularly effective against P-Selectin, and simvastatin, atorvastatin, and pitavastatin against E-Selectin. Further research is warranted to investigate whether specific statin classes and individual agents have superior capacity to reduce soluble selectin concentrations and whether this effect might be particularly beneficial in specific patient groups. Another interesting observation, in subgroup analysis, was the difference in selectin-lowering according to specific continent, with studies conducted in Europe showing a particular efficacy against P-Selectin and E-Selectin. Previous studies have investigated the concentrations of soluble selectins in different ethnic groups. In the Multi-Ethnic Study of Atherosclerosis, no significant differences in soluble E-Selectin concentrations were observed between white, black, Hispanic, and Chinese participants [105]. Other studies have also failed to detect significant differences in soluble E-Selectin and P-Selectin across ethnic groups [106,107,108]. It remains to be established whether potential ethnic-related differences in statin-mediated selectin-lowering effects might translate into different effects on surrogate markers and/or clinical endpoints in intervention trials.

The strengths of our study include the relatively large number of treatment arms analysed (41 for P-Selectin, five for L-Selectin, and 41 for E-Selectin), the assessment of possible associations between effect size and a comprehensive range of study and patient characteristics by means of meta-regression and/or subgroup analysis, and a robust assessment of the certainty of evidence according to GRADE. One significant limitation is the large-to-extreme between-study heterogeneity, which limits the generalizability of our results. However, particularly in studies investigating P-selectin, this heterogeneity was substantially attenuated in a sub-group of studies performed in Europe using atorvastatin.

## 5. Conclusions

In this systematic review and meta-analysis, treatment with statins was associated with a significant reduction in the concentrations of soluble P-Selectin, L-Selectin, and E-Selectin, a critical family of cell adhesion molecules that is involved in the pathogenesis of atherosclerosis. The selectin-lowering effect was independent of various patient and study characteristics, particularly baseline lipid profile and treatment duration, and was more prominent with specific agents, i.e., simvastatin, in studies conducted in Europe. These results warrant adequately designed intervention trials to determine whether selectin-lowering can mediate the atheroprotective effects of these agents and whether specific patient groups are more likely to benefit from this phenomenon. In particular, the reported differences in effect size according to the continent where the study was conducted require further research to determine whether ethnicity is an important mediator of the effects of statin treatment on circulating soluble selectins.

## Figures and Tables

**Table 1:** Study characteristics.

First Author, Year, Country [Reference]	N	Age (Years)	Males (%)	P-Selectinbefore Mean ± SD (ng/mL)	P-Selectinafter Mean ± SD (ng/mL)	L-Selectinbefore Mean ± SD (ng/mL)	L-Selectinafter Mean ± SD (ng/mL)	E-Selectinbefore Mean ± SD (ng/mL)	E-Selectinafter Mean ± SD (ng/mL)	Primary Condition Statin and Daily Dose Treatment Duration
Koh KK, 1999, USA [33]	28	57	0	-	-	-	-	49 ± 18	51 ± 19	Hypercholesterolaemia Simvastatin 10 mg 6 weeks
Rauch U (a), 2000, USA [34]	24	62	NR	130 ± 30	129 ± 22	753 ± 30	738 ± 31	-	-	Hypercholesterolaemia Pravastatin 40 mg 12 weeks
Rauch U (b), 2000, USA [34]	26	58	NR	105 ± 17	115 ± 15	834 ± 42	778 ± 32	-	-	Hypercholesterolaemia Simvastatin 20 mg 12 weeks
Romano M, 2000, Italy [35]	13	59	23	118 ± 63	81 ± 36	-	-	-	-	Hypercholesterolaemia Fluvastatin 80 mg 12 weeks
Sbarouni E, 2000, Greece [36]	16	66	0	-	-	-	-	17 ± 28	17 ± 30	Ischaemic heart disease Simvastatin 20 mg 8 weeks
Alonso R, 2001, Spain [37]	25	48	44	-	-	-	-	16 ± 3	18 ± 4	Familial hypercholesterolaemia Simvastatin 40–80 mg 52 weeks
Blann AD, 2001, UK [38]	17	65	59	30 ± 9	26 ± 10	-	-	-	-	Peripheral artery disease Pravastatin 40 mg 4 weeks
Sardo MA, 2001, Italy [39]	20	45	45	-	-	-	-	46 ± 12	51 ± 17	Hypercholesterolaemia Simvastatin 20 mg 24 weeks
Solheim S, 2001, Norway [40]	40	54	100	54 ± 13	52 ± 13	-	-	36 ± 8	34 ± 8	Hypercholesterolaemia Pravastatin 40 mg 8 weeks
van Haelst PL, 2001, The Netherlands [41]	10	52	90	-	-	-	-	59 ± 12	45 ± 12	Ischaemic heart disease Fluvastatin 80 mg 48 weeks
Atalar E, 2002, Turkey [42]	36	53	61	-	-	666 ± 201	584 ± 162	-	-	Hypercholesterolaemia Atorvastatin 10 mg 12 weeks
Seljeflot I (a), 2002, Norway [43]	28	NR	79	99 ± 30	86 ± 21	-	-	43 ± 19	42 ± 19	Ischaemic heart disease Atorvastatin 20 mg 12 weeks
Seljeflot I (b), 2002, Norway [43]	30	NR	93	106 ± 30	96 ± 36	-	-	49 ± 19	47 ± 21	Ischaemic heart disease Simvastatin 20 mg 12 weeks
Bogaty P, 2003, Canada [44]	17	60	6	56 ± 20	58 ± 23	-	-	74 ± 18	54 ± 14	Ischaemic heart disease Atorvastatin 10–80 mg 11 weeks
Dalla Nora I, 2003, Italy [45]	13	66	54	-	-	-	-	16 ± 3	8 ± 1	Type 2 diabetes Atorvastatin 10 mg 12 weeks
Empen K, 2003, Germany [46]	11	62	55	-	-	-	-	70 ± 37	67 ± 40	Type 2 diabetes Atorvastatin 10 mg 6 weeks
Ferroni P, 2003, Italy [47]	25	54	36	70 ± 20	52 ± 18	-	-	-	-	Hypercholesterolaemia Simvastatin 20 mg 8 weeks
Hernández C, 2003, Spain [48]	44	50	100	-	-	-	-	36 ± 4	33 ± 4	Hypercholesterolaemia Various statins and doses 16 weeks
Homma Y, 2003, Japan [49]	30	67	13	250 ± 103	196 ± 81	-	-	-	-	Type 2 hyperlipoproteinemia Fluvastatin 20–40 mg 24 weeks
Malyszko J, 2003, Poland [50]	12	NR	58	115 ± 52	84 ± 13	-	-	-	-	Kidney transplant Fluvastatin 20 mg 12 weeks
Nawawi H (a), 2003, Malaysia [51]	27	42	41	-	-	-	-	209 ± 18	35 ± 11	Familial hypercholesterolaemia Atorvastatin 80 mg 9 weeks
Nawawi H (b), 2003, Malaysia [51]	47	48	55	-	-	-	-	246 ± 14	144 ± 11	Familial hypercholesterolaemia Atorvastatin 10 mg 9 weeks
Puccetti L, 2003, Italy [52]	14	50	57	23 ± 4	10 ± 2	-	-	-	-	Hypercholesterolaemia Simvastatin 20 mg 6 weeks
van Haelst PL, 2003, The Netherlands [53]	35	42	60	-	-	-	-	69 ± 27	62 ± 27	Familial hypercholesterolaemia Simvastatin 80 mg 52 weeks
Brown SL, 2004, South Africa [54]	23	36	70	-	-	-	-	61 ± 58	61 ± 55	Familial hypercholesterolaemia Simvastatin 20–80 mg 24 weeks
Ceriello A, 2004, Italy [55]	30	54	73	-	-	-	-	79 ± 73	46 ± 55	Type 2 diabetes Simvastatin 40 mg 12 weeks
Cha JK, 2004, Korea [56]	32	60	87	97 ± 12	87 ± 8	-	-	-	-	Ischaemic stroke Simvastatin 20 mg 12 weeks
Koh KK, 2004, Korea [57]	32	62	41	-	-	-	-	44 ± 17	45 ± 20	Hypercholesterolaemia Simvastatin 20 mg 14 weeks
Malyszko J, 2004, Poland [58]	15	50	NR	103 ± 46	94 ± 36	-	-	64 ± 26	51 ± 10	Peritoneal dialysis Simvastatin 10 mg 24 weeks
Nomura S (a), 2004, Japan [59]	30	67	37	190 ± 23	164 ± 23	-	-	-	-	Hypertension and diabetes Simvastatin 10 mg 24 weeks
Nomura S (b), 2004, Japan [59]	18	64	56	108 ± 15	107 ± 15	-	-	-	-	Hypertension Simvastatin 10 mg 24 weeks
Skhra J, 2004, Czech Republic [60]	20	57	60	190 ± 54	199 ± 55	-	-	65 ± 19	70 ± 19	Type 2 diabetes Simvastatin 20 mg 12 weeks
Akçay MN, 2005, Turkey [61]	10	41	40	-	-	-	-	24 ± 3	11 ± 1	Type 2 diabetes Simvastatin 20 mg 12 weeks
Undas A, 2005, Poland [62]	20	56	70	118 ± 54	100 ± 45	-	-	-	-	Hypercholesterolaemia Simvastatin 40 mg 4 weeks
Bleske BE, 2006, USA [63]	15	56	60	30 ± 19	23 ± 20	-	-	-	-	Non-ischaemic cardiomyopathy Atorvastatin 80 mg 12 weeks
Marschang P, 2006, Austria [64]	47	59	64	92 ± 75	52 ± 27	-	-	55 ± 27	60 ± 27	Ischaemic heart disease Various statins and doses 12 weeks
Peverill RE, 2006, Australia [65]	24	59	0	43 ± 11	34 ± 9	-	-	-	-	Hypercholesterolaemia Pravastatin 20 mg 24 weeks
Potaczek PD (a), 2006, Poland [66]	10	54	NR	135 ± 63	129 ± 66	-	-	-	-	Hypercholesterolaemia Simvastatin 40 mg 4 weeks
Potaczek PD (b), 2006, Poland [66]	10	54	NR	118 ± 54	91 ± 41	-	-	-	-	Hypercholesterolaemia Simvastatin 40 mg 4 weeks
Alber HF, 2007, Austria [67]	15	57	NR	-	-	-	-	56 ± 23	52 ± 25	Ischaemic heart disease Atorvastatin 20 mg 12 weeks
Inami N (a), 2007, Japan [68]	65	65	35	177 ± 226	203 ± 282	-	-	-	-	Hypercholesterolaemia without diabetes Pitavastatin 2 mg 24 weeks
Inami N (b), 2007, Japan [68]	52	62	46	238 ± 260	200 ± 296	-	-	-	-	Hypercholesterolaemia and diabetes Pitavastatin 2 mg 24 weeks
Jeffs LS, 2007, Australia [69]	25	57	64	-	-	-	-	3.6 ± 1.5	3.9 ± 1.6	End-stage renal disease Pravastatin 10–40 mg 20 weeks
Souza-Costa DC (a), 2007, Brazil [70]	15	28	100	30 ± 16	29 ± 15	-	-	-	-	Healthy Atorvastatin 10 mg 2 weeks
Souza-Costa DC (b), 2007, Brazil [70]	15	31	100	42 ± 18	31 ± 21	-	-	-	-	Healthy Atorvastatin 10 mg 2 weeks
Barreto AC, 2008, Brazil [71]	30	35	40	40 ± 22	35 ± 16	-	-	-	-	Pulmonary arterial hypertension Rosuvastatin 10 mg 24 weeks
Bolewski A, 2008, France [72]	26	57	62	55 ± 26	54 ± 20	-	-	36 ± 20	38 ± 20	Hypercholesterolaemia Atorvastatin 20 mg 12 weeks
Del Papa N, 2008, Italy [73]	20	59	0	-	-	-	-	52 ± 89	38 ± 45	Systemic sclerosis Simvastatin 20 mg 12 weeks
Forst T, 2008, Germany [74]	80	62	48	277 ± 125	291 ± 161	-	-	-	-	High cardiovascular risk Atorvastatin 40 mg 24 weeks
Hogue JC, 2008, Canada [75]	15	55	84	-	-	-	-	50 ± 28	48 ± 22	Type 2 diabetes Atorvastatin 20 mg 6 weeks
Nomura S (a), 2008, Japan [76]	30	60	43	136 ± 33	128 ± 62	784 ± 121	769 ± 114	52 ± 10	42 ± 11	Hypercholesterolaemia Pitavastatin 2 mg 24 weeks
Nomura S (b), 2008, Japan [76]	45	62	44	238 ± 64	223 ± 33	896 ± 141	814 ± 129	74 ± 21	51 ± 10	Hypercholesterolaemia Pitavastatin 2 mg 24 weeks
Stulc T, 2008, Czech Republic [77]	27	52	30	-	-	-	-	58 ± 38	59 ± 35	Hypercholesterolemia Atorvastatin 20 mg 12 weeks
Wang L, 2008, USA [78]	25	NR	NR	111 ± 36	119 ± 71	-	-	-	-	Metabolic syndrome Simvastatin 40 mg 8 weeks
Baldassarre D, 2009, Italy [79]	85	58	85	-	-	-	-	32 ± 15	22 ± 10	Ischaemic heart disease Atorvastatin 20 mg 12 weeks
Grip O, 2009, Sweden [80]	10	32	50	255 ± 105	222 ± 41	-	-	18 ± 9	15 ± 9	Crohn’s disease Atorvastatin 80 mg 12 weeks
Nomura S (a), 2009, Japan [81]	63	61	NR	188 ± 50	182 ± 48	-	-	-	-	Hypercholesterolaemia Simvastatin 10 mg 24 weeks
Nomura S (b), 2009, Japan [81]	72	61	NR	184 ± 47	175 ± 51	-	-	-	-	Hypercholesterolaemia Pitavastatin 2 mg 24 weeks
Prázný M, 2009, Czech Republic [82]	20	57	50	203 ± 64	178 ± 51	-	-	66 ± 27	33 ± 10	Type 2 diabetes Simvastatin 20 mg 12 weeks
Serrano CV, 2009, Brazil [83]	23	63	56	-	-	-	-	46 ± 17	59 ± 24	Hypercholesterolaemia Simvastatin 40 mg 12 weeks
Fichtenbaum CJ, 2010, USA [84]	37	NR	92	61 ± 21	57 ± 13	-	-	-	-	Hypercholesterolaemia Pravastatin 40 mg 12 weeks
Kater AL, 2010, Brazil [85]	25	53	76	-	-	-	-	44 ± 20	40 ± 15	Pre-diabetes Simvastatin 20 mg 12 weeks
Kirmizis K, 2010, Greece [86]	25	63	48	-	-	-	-	77 ± 31	69 ± 31	Haemodialysis Simvastatin 10 mg 24 weeks
Xu DY, 2010, China [87]	79	64	59	101 ± 19	73 ± 15	-	-	-	-	High cardiovascular risk Atorvastatin 10 mg 8 weeks
Wu YW, 2012, Taiwan [88]	34	54	71	-	-	-	-	44 ± 50	42 ± 50	Ischaemic heart disease Atorvastatin 40 mg 12 weeks
Altun I, 2014, Turkey [89]	30	53	100	-	-	-	-	100 ± 35	89 ± 31	Acute coronary syndrome Atorvastatin 40 mg 12 weeks
Pawelczyk M, 2015, Poland [90]	31	62	57	124 ± 60	78 ± 38	-	-	-	-	Ischaemic stroke, Simvastatin 20 mg 24 weeks
Hernandez-Mijares A, 2016, Spain [91]	20	58	33	-	-	-	-	45 ± 31	39 ± 20	Hypercholesterolaemia, Simvastatin 40 mg 4 weeks
Barale C, 2018, Italy [92]	25	59	44	-	-	-	-	44 ± 15	25 ± 8	Hypercholesterolaemia Simvastatin 40 mg 8 weeks
Kotyla PJ, 2018, Poland [93]	25	55	12	67 ± 41	66 ± 26	887 ± 222	927 ± 385	276 ± 122	253 ± 125	Systemic sclerosis Simvastatin 20 mg 4 weeks

Legend: NR, not reported.

**Table 2:** The Joanna Briggs Institute critical appraisal checklist.

Study	Were the Criteria for Inclusion in the Sample Clearly Defined?	Were the Study Subjects and the Setting Described in Detail?	Was the Exposure Measured in a Valid and Reliable Way?	Were Objective, Standard Criteria Used for Measurement of the Condition?	Were Confounding Factors Identified?	Were Strategies to Deal with Confounding Factors Stated?	Were the Outcomes Measured in a Valid and Reliable Way?	Was Appropriate Statistical Analysis Used?	Risk of Bias
Koh KK [33]	Yes	Yes	Yes	Yes	Yes	Yes	Yes	Yes	Low
Rauch U [34]	Yes	Yes	Yes	Yes	No	No	Yes	No	Low
Romano M [35]	Yes	Yes	Yes	Yes	No	No	Yes	No	Low
Sbarouni E [36]	Yes	Yes	Yes	Yes	Yes	Yes	Yes	Yes	Low
Alonso R [37]	Yes	Yes	Yes	Yes	No	No	Yes	No	Low
Blann AD [38]	Yes	Yes	Yes	Yes	No	No	Yes	No	Low
Sardo MA [39]	Yes	Yes	Yes	Yes	No	No	Yes	Yes	Low
Solheim S [40]	Yes	Yes	Yes	Yes	Yes	Yes	Yes	Yes	Low
van Haelst PL [41]	Yes	Yes	Yes	Yes	No	No	Yes	No	Low
Atalar E [42]	Yes	Yes	Yes	Yes	No	No	Yes	No	Low
Seljeflot I [43]	Yes	Yes	Yes	Yes	No	No	Yes	Yes	Low
Bogaty P [44]	Yes	Yes	Yes	Yes	No	No	Yes	No	Low
Dalla Nora [45]	Yes	Yes	Yes	Yes	Yes	Yes	Yes	Yes	Low
Empen K [46]	Yes	Yes	Yes	Yes	Yes	Yes	Yes	Yes	Low
Ferroni P [47]	Yes	Yes	Yes	Yes	No	No	Yes	No	Low
Hernández C [48]	Yes	Yes	Yes	Yes	No	No	Yes	No	Low
Homma Y [49]	Yes	Yes	Yes	Yes	No	No	Yes	No	Low
Malyszko J [50]	Yes	Yes	Yes	Yes	No	No	Yes	No	Low
Nawawi H [51]	Yes	Yes	Yes	Yes	No	No	Yes	No	Low
Puccetti L [52]	Yes	Yes	Yes	Yes	No	No	Yes	No	Low
van Haelst PL [53]	Yes	Yes	Yes	Yes	No	No	Yes	No	Low
Brown SL [54]	Yes	Yes	Yes	Yes	No	No	Yes	No	Low
Ceriello A [55]	Yes	Yes	Yes	Yes	Yes	Yes	Yes	Yes	Low
Cha JK [56]	Yes	Yes	Yes	Yes	No	No	Yes	No	Low
Koh KK [57]	Yes	Yes	Yes	Yes	No	No	Yes	Yes	Low
Malyszko J [58]	Yes	Yes	Yes	Yes	No	No	Yes	No	Low
Nomura S [59]	Yes	Yes	Yes	Yes	No	No	Yes	No	Low
Skrha J [60]	Yes	Yes	Yes	Yes	No	No	Yes	No	Low
Akçay MN [61]	Yes	Yes	Yes	Yes	No	No	Yes	No	Low
Undas A [62]	Yes	Yes	Yes	Yes	No	No	Yes	No	Low
Bleske BE [63]	Yes	Yes	Yes	Yes	Yes	Yes	Yes	Yes	Low
Marschang P [64]	Yes	Yes	Yes	Yes	No	No	Yes	No	Low
Peverill RE [65]	Yes	Yes	Yes	Yes	No	No	Yes	No	Low
Potaczek PD [66]	Yes	Yes	Yes	Yes	No	No	Yes	No	Low
Alber HF [67]	Yes	Yes	Yes	Yes	No	No	Yes	No	Low
Inami N [68]	Yes	Yes	Yes	Yes	No	No	Yes	Yes	Low
Jeff LS [69]	Yes	Yes	Yes	Yes	No	No	Yes	No	Low
Souza-Costa DC [70]	Yes	Yes	Yes	Yes	No	No	Yes	No	Low
Barreto AC [71]	Yes	Yes	Yes	Yes	No	No	Yes	Yes	Low
Bolewski A [72]	Yes	Yes	Yes	Yes	No	No	Yes	No	Low
Del Papa N [73]	Yes	Yes	Yes	Yes	No	No	Yes	No	Low
Forst T [74]	Yes	Yes	Yes	Yes	No	No	Yes	No	Low
Hogue JC [75]	Yes	Yes	Yes	Yes	No	No	Yes	No	Low
Nomura S [76]	Yes	Yes	Yes	Yes	Yes	Yes	Yes	Yes	Low
Stulc T [77]	Yes	Yes	Yes	Yes	No	No	Yes	No	Low
Wang L [78]	Yes	Yes	Yes	Yes	No	No	Yes	No	Low
Baldassarre D [79]	Yes	Yes	Yes	Yes	No	No	Yes	No	Low
Grip O [80]	Yes	Yes	Yes	Yes	No	No	Yes	No	Low
Nomura S [81]	Yes	Yes	Yes	Yes	Yes	Yes	Yes	Yes	Low
Prázný M [82]	Yes	Yes	Yes	Yes	No	No	Yes	No	Low
Serrano CV [83]	Yes	Yes	Yes	Yes	No	No	Yes	No	Low
Fichtenbaum CJ [84]	Yes	Yes	Yes	Yes	No	No	Yes	No	Low
Kater AL [85]	Yes	Yes	Yes	Yes	No	No	Yes	Yes	Low
Kirmizis K [86]	Yes	Yes	Yes	Yes	No	No	Yes	Yes	Low
Xu DY [87]	Yes	Yes	Yes	Yes	No	No	Yes	No	Low
Wu YW [88]	Yes	Yes	Yes	Yes	No	No	Yes	No	Low
Altun I [89]	Yes	Yes	Yes	Yes	No	No	Yes	No	Low
Pawelczyk M [90]	Yes	Yes	Yes	Yes	No	No	Yes	No	Low
Hernandez-Mijares A [91]	Yes	Yes	Yes	Yes	No	No	Yes	No	Low
Barale C [92]	Yes	Yes	Yes	Yes	No	No	Yes	No	Low
Kotyla PJ [93]	Yes	Yes	Yes	Yes	No	No	Yes	No	Low

## Data Availability

The data that support the findings of this systematic review and meta-analysis are available from the first author, A.Z., upon reasonable request.

## Supplementary Materials

The following are available online at https://www.mdpi.com/article/10.3390/biomedicines9111707/s1, Table S1: PRISMA 2020 abstract checklist; Table S2: PRISMA 2020 checklist and search strategy.
